# Historical effects of dissolved organic carbon export and land management decisions on the watershed-scale forest carbon budget of a coastal British Columbia Douglas-fir-dominated landscape

**DOI:** 10.1186/s13021-017-0083-z

**Published:** 2017-07-14

**Authors:** B. P. Smiley, J. A. Trofymow

**Affiliations:** 1 Natural Resources Canada, Canadian Forest Service, 506 West Burnside Road, Victoria, BC V8Z 1M5 Canada; 2 Biology Department, University of Victoria, Victoria, BC V8W 3R4 Canada

**Keywords:** Carbon budgets, Deforestation, Dissolved organic carbon, Disturbance history, CBM-CFS3

## Abstract

**Background:**

To address how natural disturbance, forest harvest, and deforestation from reservoir creation affect landscape-level carbon (C) budgets, a retrospective C budget for the 8500 ha Sooke Lake Watershed (SLW) from 1911 to 2012 was developed using historical spatial inventory and disturbance data. To simulate forest C dynamics, data was input into a spatially-explicit version of the Carbon Budget Model-Canadian Forest Sector (CBM-CFS3). Transfers of terrestrial C to inland aquatic environments need to be considered to better capture the watershed scale C balance. Using dissolved organic C (DOC) and stream flow measurements from three SLW catchments, DOC load into the reservoir was derived for a 17-year period. C stocks and stock changes between a baseline and two alternative management scenarios were compared to understand the relative impact of successive reservoir expansions and sustained harvest activity over the 100-year period.

**Results:**

Dissolved organic C flux for the three catchments ranged from 0.017 to 0.057 Mg C ha^−1^ year^−1^. Constraining CBM-CFS3 to observed DOC loads required parameterization of humified soil C losses of 2.5, 5.5, and 6.5%. Scaled to the watershed and assuming none of the exported terrestrial DOC was respired to CO_2_, we hypothesize that over 100 years up to 30,657 Mg C may have been available for sequestration in sediment. By 2012, deforestation due to reservoir creation/expansion resulted in the watershed forest lands sequestering 14 Mg C ha^−1^ less than without reservoir expansion. Sustained harvest activity had a substantially greater impact, reducing forest C stores by 93 Mg C ha^−1^ by 2012. However approximately half of the C exported as merchantable wood during logging (~176,000 Mg C) may remain in harvested wood products, reducing the cumulative impact of forestry activity from 93 to 71 Mg C ha^−1^.

**Conclusions:**

Dissolved organic C flux from temperate forest ecosystems is a small but persistent C flux which may have long term implications for C storage in inland aquatic systems. This is a first step integrating fluvial transport of C into a forest carbon model by parameterizing DOC flux from soil C pools. While deforestation related to successive reservoir expansions did impact the watershed-scale C budget, over multi-decadal time periods, sustained harvest activity was more influential.

**Electronic supplementary material:**

The online version of this article (doi:10.1186/s13021-017-0083-z) contains supplementary material, which is available to authorized users.

## Background

Climate change mitigation requires a global effort to reduce the amount of greenhouse gases (GHG) in the atmosphere. Strategies to decrease atmospheric concentrations of CO_2_ require both reduction of anthropogenic emissions and improved means of C sequestration. The potential of forests in Canada to be net C sinks, while highly variable in space and time [1], can be considered to have a positive role in climate change mitigation. In temperate and boreal forests, while the natural disturbance regime is a primary driver of the ecosystem C balance, forest management activities also have an impact [2]. If forest management practices are amended to include C sequestration, management practices can be optimized to allow for the forested land base to sequester and store more C than it would have otherwise [3]. This can be accomplished through various management practices, including forest conservation in parks and protected areas [4], enhanced silviculture and harvest optimization [3] and longer-lived harvested wood products that displace more C intensive products [5].

The movement of C from the forested terrestrial system into the aquatic system is a subtle feature of the C cycle that has not been widely included in modelling efforts, dissolved organic C being a primary vector for C transport between these systems. Globally, human use of the terrestrial land base has increased the transfer of C to inland aquatic systems by as much as 1.0 pentagrams of C per year [6]. At the watershed-scale, accounting for the export of terrestrial C via fluvial systems is necessary when evaluating the C storage effect of different forest management practices. Anthropogenic disturbance can also have a considerable impact on the transport of suspended sediments, 90% of which do not make it to the ocean and deposit in lake and floodplain sediments [7]. Carbon burial in lake sediment is an important component of watershed-scale C budgets and has unique implications for areas managed for water supply [8, 9].

While the link between major hydrological events within a watershed and C being discharged in fluvial systems from that watershed are highly correlated, other watershed characteristics that may impact the concentration of C fluxes have not been well studied [10]. Dissolved organic matter, or dissolved organic carbon (DOC) as it commonly measured, is sourced from leached decaying plant material and mineral soil layers [11]. The fraction of lakes and wetlands within a catchment is known to be an important regulator of DOC export [12]. While the presence of bogs or wetlands within a catchment is a major source of DOC [11], natural or anthropogenic disturbance to forest cover and other land use classes [13] can also greatly influence the type and amount of C being exported from the terrestrial component of a watershed [10, 11, 14]. Forest cover disturbance affect both the short term discharge of DOC to the aquatic system due to factors such as amplified overland water flow [15] and rapid accumulation of organic matter [16], but also long term DOC discharge resulting from slow redevelopment of forest floor and soil C pools.

Research by Creed et al. [17] indicates that in North America, both environmental factors (summer precipitation, water residence time) and ecological factors (forest type and age) need to be considered when attempting to increase resilience of forested water supply watersheds against future climate warming. Considering, in the twentieth century, the area of inland river systems in the form of reservoirs increased by approximately 700% [18], the lateral transport of C from terrestrial systems to inland aquatic environments represents a significant C flux that may be altered by future climate change through increased sudden rainfall events and longer periods of summer drought [19]. Without understanding the existing magnitude of this C flux, the potential impact on watershed-scale C budgets is largely unknown.

The Carbon Budget Model of the Canadian Forest Sector 3 (CBM-CFS3) has been used in a C accounting and reporting capacity in numerous operational, regional and national scale analyses, both in Canada and internationally [20]. The model has also been used to evaluate the effectiveness of forest management strategies to mitigate climate change [3]. Recent work modelled carbon stocks and fluxes using spatially-explicit forest inventory and remotely-sensed disturbance datasets with a version of CBM-CFS3 that processes and outputs spatial layers [21, 22]. Within these analyses, the model assumes that any C transfers out of the forest system via dissolved C are included in decomposition releases to the atmosphere [20]. While small relative to the land–atmosphere exchange of C, the land-inland aquatic system exchange of C may account for a significant proportion of C that is generally assumed to be respired to the atmosphere in modeling efforts or remain within land ecosystems [23].

**Fig. 1:**
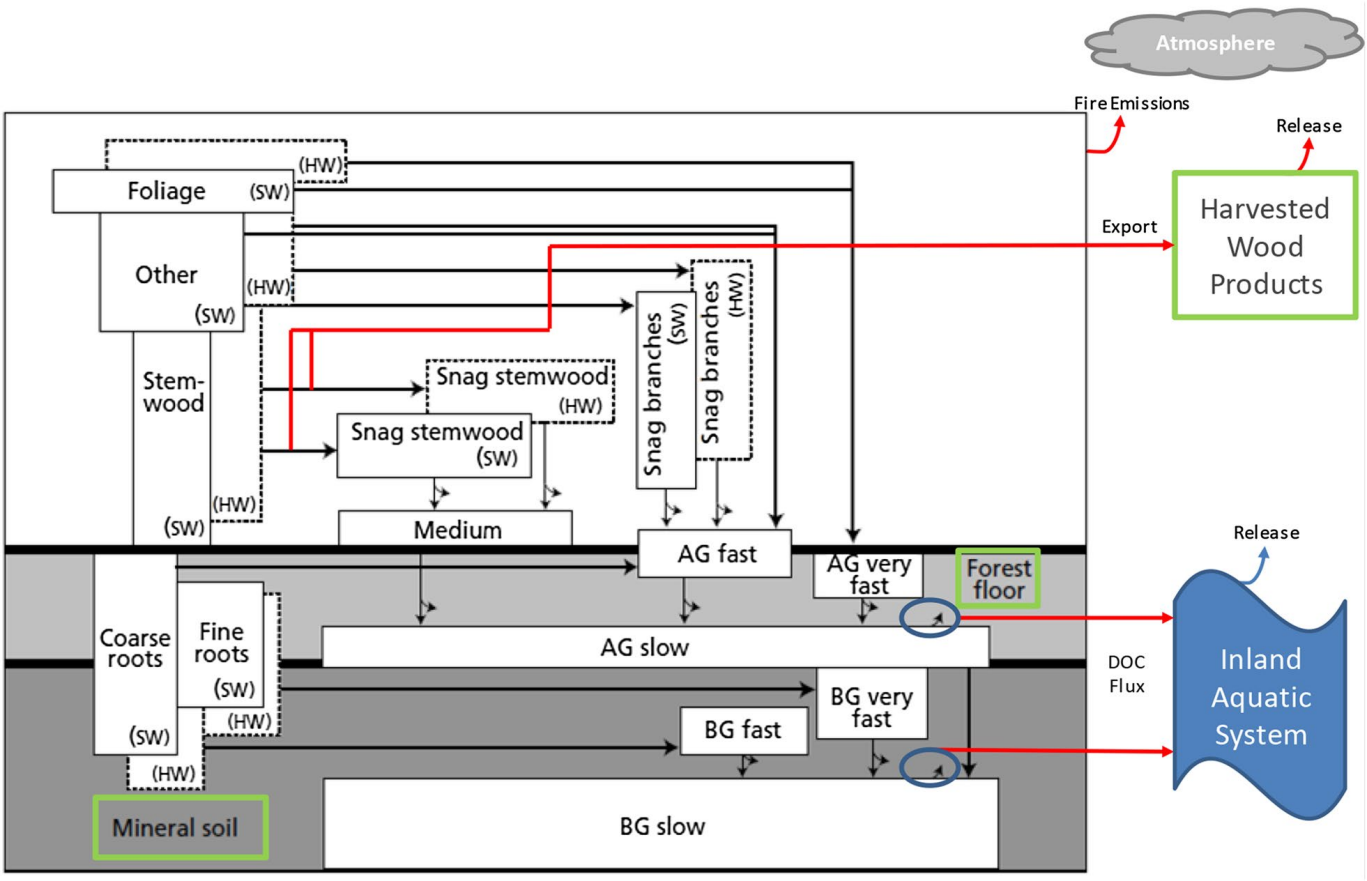
CBM-CFS3 carbon pool structure augmented to include transfers of C from the aboveground slow and belowground slow pools to the inland aquatic system via dissolved organic C (DOC) (Adapted from [36])

**Fig. 2:**
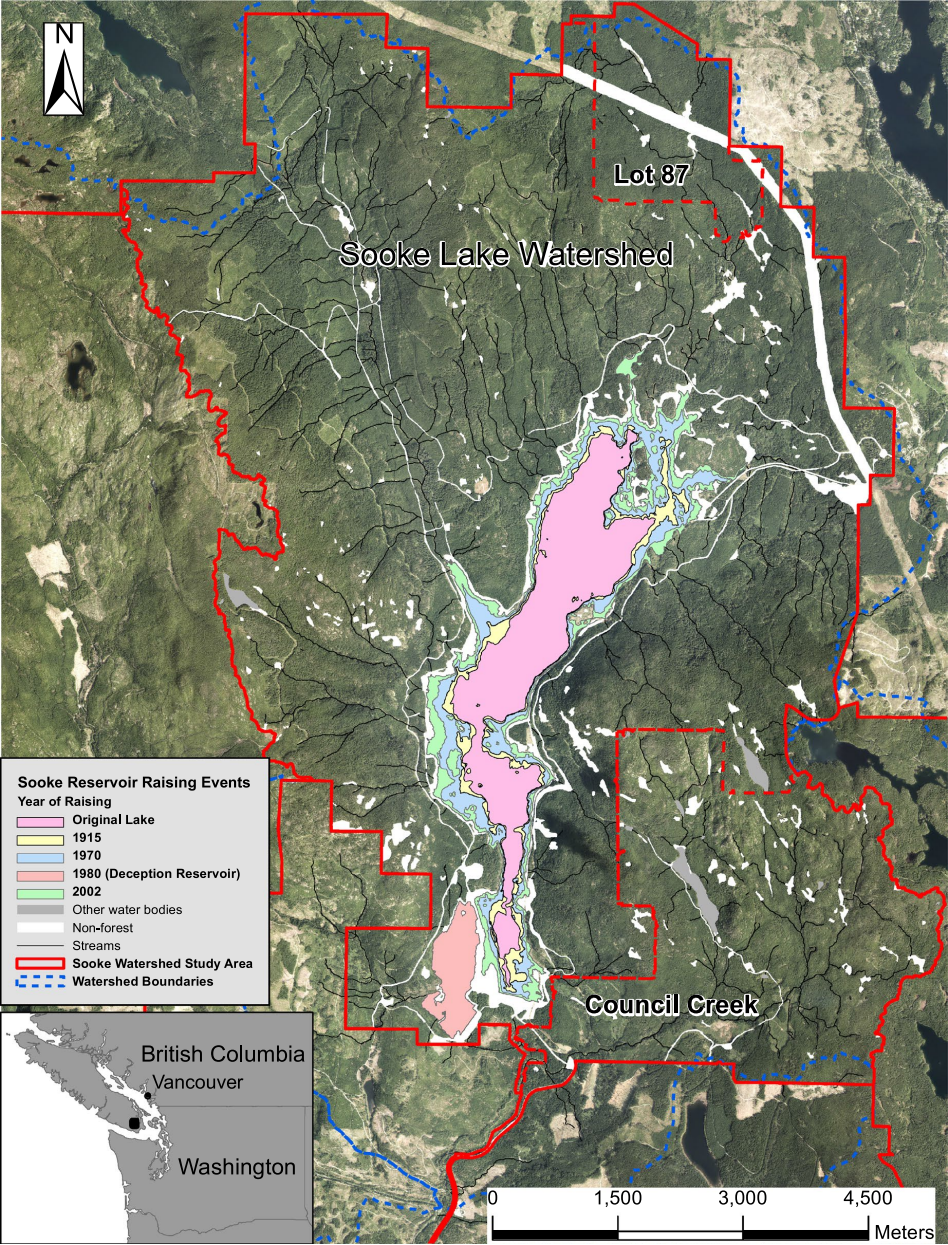
Sooke Lake Watershed study area

The main purpose of this study was to address a gap in current forest C budget research relating to the relative importance of including DOC as a dynamic C export mechanism from the terrestrial ecosystem. The specific objectives of this study were to: (1) parameterize the CBM-CFS3 modeled transfer of C from the terrestrial to the inland aquatic system using [DOC] and stream discharge data from 1996 to 2012 (Fig. 1), (2) apply the DOC parameterization to the Sooke Lake Watershed to estimate the impact on landscape C budgets over 100 years (1911–2012), and (3) compare the relative impacts of land management activities from reservoir creation and expansion and sustained harvest activity on the landscape C budget.

## Methods

### Study area

The Sooke Lake Watershed (SLW) Reservoir (48°31′30″N, 123°37′30″W) is located on southern Vancouver Island, British Columbia (BC), Canada (Fig. 2). The SLW, part of the Greater Victoria Water Supply Area, is approximately 40 km north of Victoria and is 8595 ha in size of which 810 ha is now reservoir. The Capital Regional District (CRD) ownership of the Sooke Lake water supply area constitutes over 90% of the area that drains into Sooke reservoir [24].

The SLW lies within the Nanaimo Lowlands Physiographic region and is dominated by the Coastal Western Hemlock, Very Dry Maritime biogeoclimatic zone [25]. It is a mild and moist climate with approximately 1640 mm of mean annual precipitation and warm dry summers with an average July air temperature of 16.4 °C. The wet season spans October to March and is characterized by a large hydrograph peak in the late fall followed by consistent rainfall for the remainder of the season until spring [26]. The winters are mild and typically free of extended sub-zero temperatures. During the winter some snowpack does exist in the watershed [27]. By April, precipitation begins to taper off; June has the least variable precipitation regime while July and August experience maximum temperatures and minimum precipitation [26].

Unlike the majority (95%) of forest land in BC which is in crown (public) possession [28], the SLW and adjacent areas became private land as part of the Esquimalt and Nanaimo Railway land grant in 1884. The majority (80%) of the SLW was bought and managed for Victoria’s water supply in 1911 by the Greater Victoria Water District, now the CRD. Due to the potential negative implications of the remaining 20% of lands within the watershed being managed without consideration for water quality, the CRD, through a combination of land exchanges and purchases, eventually acquired much of the remaining lands within the SLW. Including the Council Lake drainage that is diverted into Sooke Reservoir, approximately 98% of the area that drains into Sooke Reservoir is now CRD-owned.

Data on forest disturbances in the SLW were consolidated into a geodatabase for the period 1911–2012 [21]. Sooke Lake was dammed for Greater Victoria’s water supply (1915) and the reservoir system was expanded three times (1970, 1980, and 2002). The SLW experienced three distinct management periods. Until the mid-1950s, very few disturbances occurred in areas owned and managed for water supply. Conversely, Council Creek catchment and Lot 87 (Fig. 2) were owned by logging companies until the 1990s and were intensively harvested during the 1920s and 1930s. Beginning in the 1950s, a period of sustained harvest activity began within areas owned for water supply and lasted until the mid-1990s. Harvesting then ceased and the SLW in its entirely, owned and managed fully for water supply, experience no further stand-replacing disturbances, other than those associated with the reservoir expansion of 2002.

### Gauged catchments

The catchments of the three gauged creeks of Rithet, Judge, and Council within the SLW constitute 44% of the total watershed area (Table 1). Rithet is the largest and only catchment with perennial stream flow and consequently is the largest contributor of water to the reservoir. On average, Rithet catchment is the steepest at 17°, and has the largest range of elevation, from 188 m at lakeside to 840 m (average elevation is 450 m). Sustained yield forestry occurred in the Rithet valley between 1954 and 1996, harvesting high quality old growth (>250 years) Coastal Douglas-fir stands. Yet, of the three gauged catchments, Rithet has the highest proportion of forest considered to be mature forest (≥80 years) at 67% (Table 2) and has the least extensive disturbances over the last 100 years. Due to the low proportion of both lakes and wetlands, Rithet catchment has limited capability to buffer stream discharge or alter constituent loading once the runoff enters Rithet Creek.

**Table 1:** Individual catchment (Rithet, Council, Judge) and landscape units (Rithet + Rithet-like, Council + Council-like, Judge + Judge-like) sharing similar physiographic and hydrologic characteristics [29] for scaling up to SLW level of analysis

Catchment	Area (ha)	% of SLW
Rithet	1824.9	21.2
Council	1189.4	13.8
Judge	765.1	8.9
Rithet + Rithet-like	3926.4	45.7
Council + Council-like	1473.2	17.1
Judge + Judge-like	2822.5	32.8
Not modelled (non-forest)	373.1	4.3
SLW total	8595.1

**Table 2:** Individual catchment (Rithet, Council, Judge) and landscape unit (Rithet + Rithet-Like, Council + Council-Like, Judge + Judge-Like) characteristics in 2012 including areas of forest seral stage, wetlands and lakes (total area and percent of catchment)

Catchment	Rithet	Council	Judge	Rithet + Rithet-like	Council + Council-like	Judge + Judge-like
Immature forest^a^						
Area (ha)	601.8	899.2	322.6	1353.8	1007.2	1150.3
% of catchment	33.2	78.7	43.5	36.6	71.4	46.5
Mature forest^a^						
Area (ha)	1210.8	243.0	418.6	2347.2	403.3	1325.4
% of catchment	66.8	21.3	56.5	63.4	28.6	53.5
Wetlands						
Area (ha)	7.8	15.3	23.5	22.5	16.3	59.8
% of catchment	0.4	1.3	3.1	0.6	1.1	2.1
Lakes						
Area (ha)	0.8	16.1	0.0	14.6	16.1	1.0
% of catchment	0.0	1.4	0.0	0.4	1.1	0.0

^a^Immature forests are considered to be stands less than 80 years while mature forests are equal to or older than 80 years

In contrast to Rithet, the Council catchment has had an intense and distributed disturbance history, spanning from the 1930s through the 1990s and has the highest proportion of juvenile and immature forest (<80 years) at 79% (Table 2). Council has roughly the same mean slope (16.5°) and elevation (450 m) as Rithet, yet Council has a much lower peak elevation (630 m). Council catchment contains a 14 ha lake into which the majority of the catchment drains before exiting into Council Creek. This hydrologic feature has important implications for constituent flux from the terrestrial land base of Council to Sooke Reservoir.

Judge Creek is the most northern and smallest of the three catchments (Table 1). The disturbance history of Judge is characterized by a short period of intense clear-cut logging and broadcast burning during the late 1920s. Other areas of Judge were harvested from the early 1950s until the mid-1980s and by 2012 56% of the catchment was considered to be mature and 44% was immature forest (Table 2). The most pronounced physiographic and hydrologic difference between Judge and the other catchments is the prevalence of relatively large wetland areas. Judge catchment has the lowest proportion of area covered by lakes/ponds, yet over 3% of Judge land cover is considered wetlands compared to Rithet’s 0.5% and Council’s 1.3%. These wetlands are contiguous with the drainage in Judge Creek and thus have a significant impact on the load of dissolved stream constituents into Sooke Reservoir.

### Catchment scale analyses

#### Hydrological data

Quarter-hourly stream discharge measurements from January 1st 1996 to December 31st 2012 for the, Rithet, Judge, and Council catchments were supplied by the CRD (J. Blaney, personal. communication). The diversion from the Council catchment makes up 90% of the combined Council-Trestle discharge where stream flow is measured using a mechanical totalizer. Both Rithet and Judge catchments use a concrete weir and water level recording device to determine stream discharge [29] (F. Hall, personal. communication).

DOC concentration (mg/L) was taken intermittently between 1997 and 2008 at the Rithet, Judge and Council outflow points into Sooke reservoir. 50 ml water samples were collected using either a Sutek sampler or sampling rod close to the water surface and transported to the CRD lab in a cooler (J. Blaney, personal. communication) and a Shimadzu TOC analyzer used to determine DOC (<0.45 μm) in the sample.

#### Software

The ‘R’ environment [30] and related time series package (i.e. zoo package) were used to merge stream flow and DOC measurement data files into an acceptable format to process into daily values for further analysis. The R package rLOADEST [31] derived from the FORTRAN Load Estimator (LOADEST) program was used to estimate annual DOC loads from concentration and stream flow measurements [32]. While three statistical estimation methods are available in LOADEST, for the purposes of this study, adjusted maximum likelihood estimation (AMLE) was used (Eq. 1). Instantaneous load estimates are derived from all observation in the estimation dataset using:

1 $$ \hat{L}_{AMLE} = exp \left( {a_{0} + \sum\limits_{j = 1}^{M} {a_{j} X_{j} } } \right)H(a,b,s^{2} ,\alpha ,\kappa ) $$

where $\hat{L}_{AMLE}$ is the AMLE instantaneous load estimate, *a* and *b* are explanatory variable functions, *α* and *κ* are gamma distribution parameters and *s* ^2^ is the residual variance [32–34]. AMLE allows for a “nearly unbiased” estimation of instantaneous dissolved stream constituent load [33]. Of the other two methods, maximum likelihood estimation (MLE) is more commonly used when the observation data set is uncensored (no observation concentration less than the laboratory detection limit) and least absolute deviation (LAD) when model residuals are not normally distributed, both of which were not the case with this study [32].

rLOADEST provides both a collection of predefined models that can be selected based on the ‘best fit’ with the data, and the ability for the user to define a unique model form. In this case, ‘best fit’ is defined as the lowest Akaike information criterion (AIC). AICc (c for correction) is an extension of AIC that corrects for small sample size by including an ‘effective sample size’ variable (n). Model coefficients are developed using ordinary least squares (OLS) regression. This regression equation (Eq. 2) is then used to calculate estimates of log- load for each observation in the time series. The full form of AICc is:

2 $$ {\text{AICc}} = - 2\left( {{ \log } - {\text{likelihood}}} \right) + 2{\text{K}} + \frac{2K(K + 1)}{(n - K - 1)} $$

where K is the number of estimated parameters included in the model and *n* is the effective sample size.

#### Empirical DOC load estimation and application

Annual DOC load reconstructions for Rithet, Judge and Council were determined for the period 1996–2012 (calibration period). Chemical concentration data are often scarce compared to measurements of stream flow. However, through development of a regression relationship (for model form see Eq. 3), missing concentration data were interpolated using available site-specific concentration and stream discharge measurements.

The model form used was:

3 $$ Instantaneous Load = a_{0} + a_{1} \ln Q a_{2} \sin \left( {2\pi dtime} \right) + a_{3} \cos \left( {2\pi dtime} \right) $$

where ln*Q* = ln(stream flow) − center of ln(stream flow); *dtime* = decimal time − center of decimal time and *a*_0_ to *a*_3_ are model coefficients.

Once the regression model form was defined, various temporal scales of DOC load and concentration were predicted to interrogate the output data. Daily DOC concentration and flow values for the three catchments were examined in relation to measured DOC concentrations in order to gauge the model’s ability to interpolate concentration at the daily temporal scale. For CBM-CFS3 parameterization, annual DOC load values were required; therefore calendar year DOC load in Mg C per day were exported from rLOADEST. These are considered to be the ‘observed’ values (see Additional file 1: Figure S1 for daily measured and empirically fit DOC values). As allochthonous carbon (i.e., the terrestrial environment) is the primary source of carbon for most small streams [35], the in-stream DOC load was used as a surrogate for DOC flux from the CBM-CFS3 soil C pools. Therefore the DOC load values were annualized to Mg C per year and converted to a unit area value given the area of each catchment of interest for use in parameterizing CBM-CFS3 DOC fluxes.

### Watershed scale DOC fluxes and baseline C budget using CBM-CFS3

#### CBM-CFS3

Smiley et al. [21] describes the use of CBM-CFS3 in a fully spatial mode and the development of the retrospective C budget for the SLW; a brief description of the model function follows. CBM-CFS3 runs on annual time-steps and uses growth and yield curves and forest cover inventory attributes to estimate stand- and landscape-level biomass C dynamics [36]. The model estimates annual biomass turnover (e.g., litter fall) which then flows to detrital C pools each of which have varying temperature-dependant decay rates based on the type of plant material represented. Carbon from decaying plant material is either lost as CO_2_ to the atmosphere or is transferred to humified soil C pools with slow decay rates released from those pools, by default, as CO_2_. Using this pool structure, the model accounts for C stocks and stock changes in tree biomass and dead organic matter [36]. The model can assess past changes in C stocks by using management and disturbance information as well as evaluate future changes that might result from modified management schemes or altered disturbances patterns [35].

As a forest-sector model, CBM-CFS3 only simulates C pools for the forested areas of a landscape. Gaseous C fluxes occur between the terrestrial system and the atmosphere while forest harvesting results in a C export from the ecosystem as round wood. Integration of terrestrial-to- aquatic C fluxes in CBM-CFS3 occur through the fraction of the slow aboveground and slow belowground dead organic matter (DOM) C pools that respire to the atmosphere. The aboveground slow DOM pool includes the F, H, and O soil horizons and corresponds to the ‘Litter’ pool in the IPCC good practice guidance (GPG) [37]. These horizons include humified organic matter that develop from the decomposition of litter and woody material [38]. The belowground slow DOM corresponds to a segment of the “soil organic matter” GPG pool and specifically includes humified organic matter in the mineral soil layer [20]. Heterotrophic respiration from these slow pools is dependent on the annual base decay rate, 1.5% per year for the aboveground slow DOM pool and 0.33% per year for the belowground slow DOM pool [20]. The path by which decaying slow C exits the terrestrial system is determined by the “fraction to atmosphere” parameters which have a default value of 1, (i.e., 100% of respired C goes to the atmosphere). Adjusting the default value to less than 1 result in a fraction of the C exported from the forest system as DOC.

#### Watershed-scale DOC fluxes

The per hectare observed DOC load values were used to calibrate CBM-CFS3 model runs for the Rithet, Council and Judge catchments to partition the decay losses from the slow aboveground and slow belowground DOM C pools to either DOC flux or CO_2_ to the atmosphere. This DOC fraction parameter was calibrated so that modelled annual ha^−1^ DOC flux matched the observed DOC loads for each catchment separately. High variance in observed annual DOC loads was most likely a result of higher or lower stream flow years; the mean DOC load for each catchment for the 1996–2012 period were used to adjust the DOC fraction parameters. Through multiple iterations of CBM-CFS3 runs, the aboveground and belowground DOC fraction parameter values were calibrated by first adjusting the parameter values prior to a model run, comparing the annual modelled DOC fluxes for the 1996–2012 period to the observed mean DOC load values, then repeating until modelled DOC flux ha^−1^ for each catchment was within 0.001 Mg C ha^−1^ of the observed DOC load. The remaining SLW catchments were then assigned DOC fraction parameter values based on the physiographic and hydrologic similarities of the 35 ungauged to the three gauged catchments as defined by Werner [29]. A model run was then conducted on the entire SLW using the same disturbance history as the existing retrospective C budget [22] using the calibrated DOC fraction parameters for all catchments.

### Land management scenarios

Alternative historic land management scenarios were conducted to allow for the quantification and direct comparison of the effects that different land management decisions had on the C budget of the land base over an extended timeframe. Alternative management scenarios were only applied to areas that were owned and operated by the CRD for the entire study period (80%). The conditions for the baseline and alternative management scenarios include:

- *Baseline—*disturbance and management history as occurred from 1911 to 2012 and as described in the “Study area” section
- *Scenario #1 (SC1)*—*water supply without deforestation or forest management*—no forest harvesting or reservoir raising (flooding) between 1911 and 2012 within the original ownership boundary (disturbances in Lot 87 and Kapoor land maintained)
- *Scenario #2 (SC2)*—*water supply without forest management* **—**reservoirs are created and raised as in baseline model runs, however, no forest harvesting occurs between 1911 and 2012 within the original ownership boundary (disturbances in Lot 87 and Kapoor land maintained).

The SC1 scenario represents a situation where the SLW was left in its original state and supposes that the Greater Victoria demand for water could be entirely supplied by the original Sooke Lake. No reservoir expansion or logging occurs within the original CRD tenure.

In the baseline and SC2 scenario, observed reservoir expansion required to meet water demand were maintained. However, the capital projects necessary for these events, including land clearing, engineering and dam construction, are assumed to be financed by means other than logging revenue and therefore these disturbances do not take place. This management regime mimics that which has been in place since the mid-1990s whereby population increases in Greater Victoria have occurred (and are incorporated into future plans) but forestry activity within the water supply area has ceased.

For both SC1 and SC2, disturbances related to natural events, adjacent lands or transportation access were preserved within the CRD tenure. These included wildfire and insect outbreaks, transportation corridor clearing for railway, access road and transmission line right-of-ways and escaped fires from adjacent lands.

Table 3 shows the breakdown of forest and non-forest areas between the Baseline, SC1 and SC2 scenarios. By comparing the difference in cumulative net biome production (ƩNBP) for SC1 vs SC2 scenarios (for which neither experience forest management on CRD ownership lands but the latter includes deforestation due to reservoir creation) the C budget consequences of just the deforestation events can be investigated. Comparing the difference in ƩNBP for Baseline vs SC2 scenarios (for which both experience reservoir expansion but the latter includes no forest management) examines the consequences of forestry activities alone.

**Table 3:** Area of analysis units and non-forest in 1911 and baseline, scenario 1 and scenario 2 in 2012

Analysis unit	Description	Area in 1911 (ha)	Area in 2012 (ha)		
			Baseline	SC1	SC2
Productive forested land					
1	Fir	5371	4326	5209	4830
2	Fir–cedar	1048	760	938	964
3	Fir–hemlock/Grand fir/Sitka Spruce	1097	1479	1182	1075
4	Fir–alder/maple/poplar/arbutus	9	357	75	57
5	Cedar leading with conifer mix	35	48	61	55
6	Hemlock	5	6	11	6
7	Hemlock–Fir	217	247	276	258
8	Hemlock–cedar	33	34	34	43
9	Broadleaf greater than 75% composition	8	20	33	34
10	Alder–conifer mix	18	13	14	14
Total		7841	7290	7833	7336
Non-forest land					
Sooke Lake/reservoir		373 (49%)	813 (62%)	373 (49%)	813 (65%)
Other un-established/non-forest land		382 (51%)	492 (38%)	389 (51%)	446 (35%)
Total		754	1305^a^	762	1259

^a^80% of the change in non-forest land is due to reservoir creation, the remainder is from road/railway creation, etc.

### Fate of exported round wood

Under IPCC guidelines in 2012, the existing assumption within CBM-CFS3 is that round wood C exported to wood products is immediately released to the atmosphere as CO_2_ at the time of harvest [37]. More recent guidelines allow for tracking the fate of round wood in harvest wood products (HWP) using separate models [39]. An alternative British Columbia-specific framework has been developed to run in conjunction with CBM-CFS3 C pool and flow capabilities (CBMF-HWP) [5]. The HWP model tracks the C storage of wood products post-harvest by simulating primary milling, construction and secondary manufacturing, retirement from material in-use, and disposal and decay of forest products. Once considered “in-use”, the C is stored between 2 and 90 years, depending on the half-live of the pool (single family homes being the longest and shipping products being the shortest) [5].

While our analysis does not explicitly track disposal and decay of HWP over time, we investigated the implications of harvested round wood between the alternative management scenarios. The simple emissions factor for whitewood from Dymond [5] (0.52) was adjusted (0.58) to include emissions associated with outside-bark dimensions (i.e. round wood) exported from CBM-CFS3.

## Results

### DOC fluxes

#### Catchment scale

**Fig. 3:**
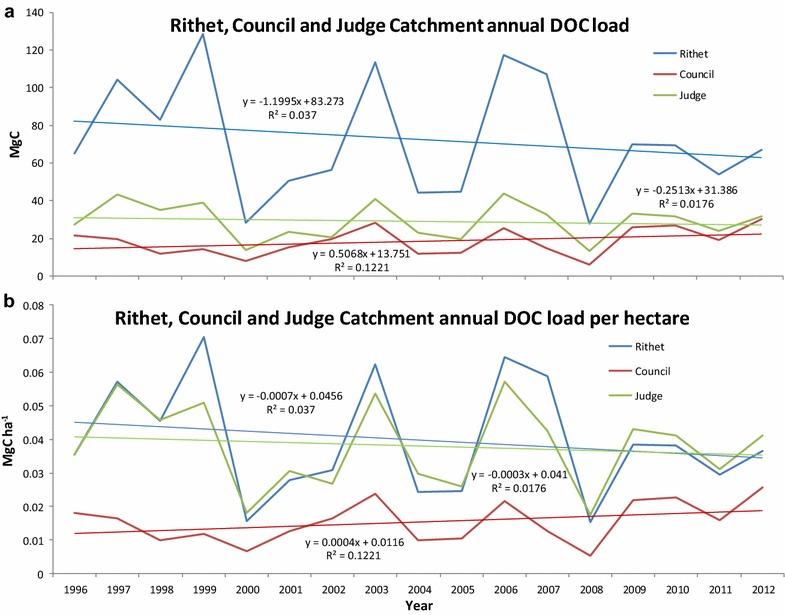
Dissolved organic carbon (DOC) load and trendlines from 1996–2012 on a total DOC flux yr^−1^ (**a**) and DOC flux ha^−1^ yr^−1^ (**b**) basis

Over the course of the calibration period (1996–2012) mean DOC load from the Rithet catchment was approximately 72.5 and 29.1 Mg C year^−1^ for judge. Council exported the lowest on average of 18.3 Mg C year^−1^ to DOC (Fig. 3a). As Council is the second largest catchment by area, on a per hectare basis, mean annual DOC load was significantly lower (0.0154 Mg C ha^−1^ year^−1^) than the other two catchments (0.0397 Mg C ha^−1^ year^−1^ for Rithet vs 0.0381 Mg C ha^−1^ year^−1^ for judge) (Fig. 3b). In all three catchments the annual variability in DOC load is closely tied to the annual stream flow. This is most likely due to the higher positive correlation between DOC load and stream flow as opposed to DOC concentration. Over the course of the study period Council was the only catchment that showed an upward trend in DOC flux, while Judge and Rithet trended downward slightly; however, the trends for all three catchments were not significant (Fig. 3). As the observed DOC load magnitudes varied spatially among the three catchments, the CBM-CFS3-model parameters of DOC flux from the slow aboveground and belowground DOM pools required different DOC fractions.

After model calibration, the three unique fraction-to-atmosphere parameters derived for Rithet, Council and Judge catchments were 0.945, 0.975, and 0.935, respectively (Table 4). The fraction-to-atmosphere parameter from the slow belowground pool remained constant at 0.99 for all three catchments. Carbon in the slow aboveground DOM pool is more labile relative to belowground DOM owing to the constituents that are represented within it [16]. Carbon from the aboveground DOM pool was considered to have a greater fraction of labile C exported as DOC. This supposition was based on a higher probability of aboveground DOM in overland flow, and it reaching a watercourse. Some of this C is transferred to the slow belowground pool, and is represented in the model as a 0.6% annual transfer. In contrast, DOC from the relatively large belowground slow DOM component was considered less mobile and that microbes consumed more than 90% before it could enter a watercourse [40]. As a result, a DOC fraction value of 1% was considered appropriate for the belowground slow DOM pool.

**Table 4:** Calibrated CBM-CS3 parameters partitioning C losses from decaying slow aboveground (AG) and belowground (BG) DOM pools to the atmosphere (fraction to atmosphere) or as dissolved organic carbon (DOC) (fraction to DOC)—modelled and observed values of mean and mean ha^−1^ Mg of carbon 1996–2012 for Rithet, Judge and Council catchments used to derive parameter values

Catchment	Slow DOM pool	Fraction to atmosphere	Fraction to DOC	Modelled value		Observed value	
				17 year mean (Mg C year^−1^)	17 year mean (Mg C ha^−1^ year^−1^)	17 year mean (Mg C year^−1^)	17 year mean (Mg C ha^−1^ year^−1^)
Rithet	AG	0.945	0.055	60.4	0.0331		
	BG	0.99	0.01	11.9	0.0065		
	Total			72.4	0.0397	72.5	0.0397
Council	AG	0.975	0.025	11.4	0.0096		
	BG	0.99	0.01	7.4	0.0062		
	Total			18.7	0.0157	18.3	0.0154
Judge	AG	0.935	0.065	23.4	0.0306		
	BG	0.99	0.01	5.6	0.0073		
	Total			29.0	0.0379	29.1	0.0381

Judge catchment had high per hectare DOC flux relative to the size of the slow aboveground DOM pool, consequently resulting in the largest DOC fraction parameter at 6.5%. The DOC fraction parameter was 5.5% for Rithet and 2.5% for Council. The relative size of the modelled slow aboveground DOM pools over the course of the study period [Rithet (63 Mg C ha^−1^ ±1) > Judge (49 Mg C ha^−1^ ±1) > Council (41 Mg C ha^−1^ ±2) ranked similarly to the observed DOC load for all three catchments.

#### Watershed scale

**Fig. 4:**
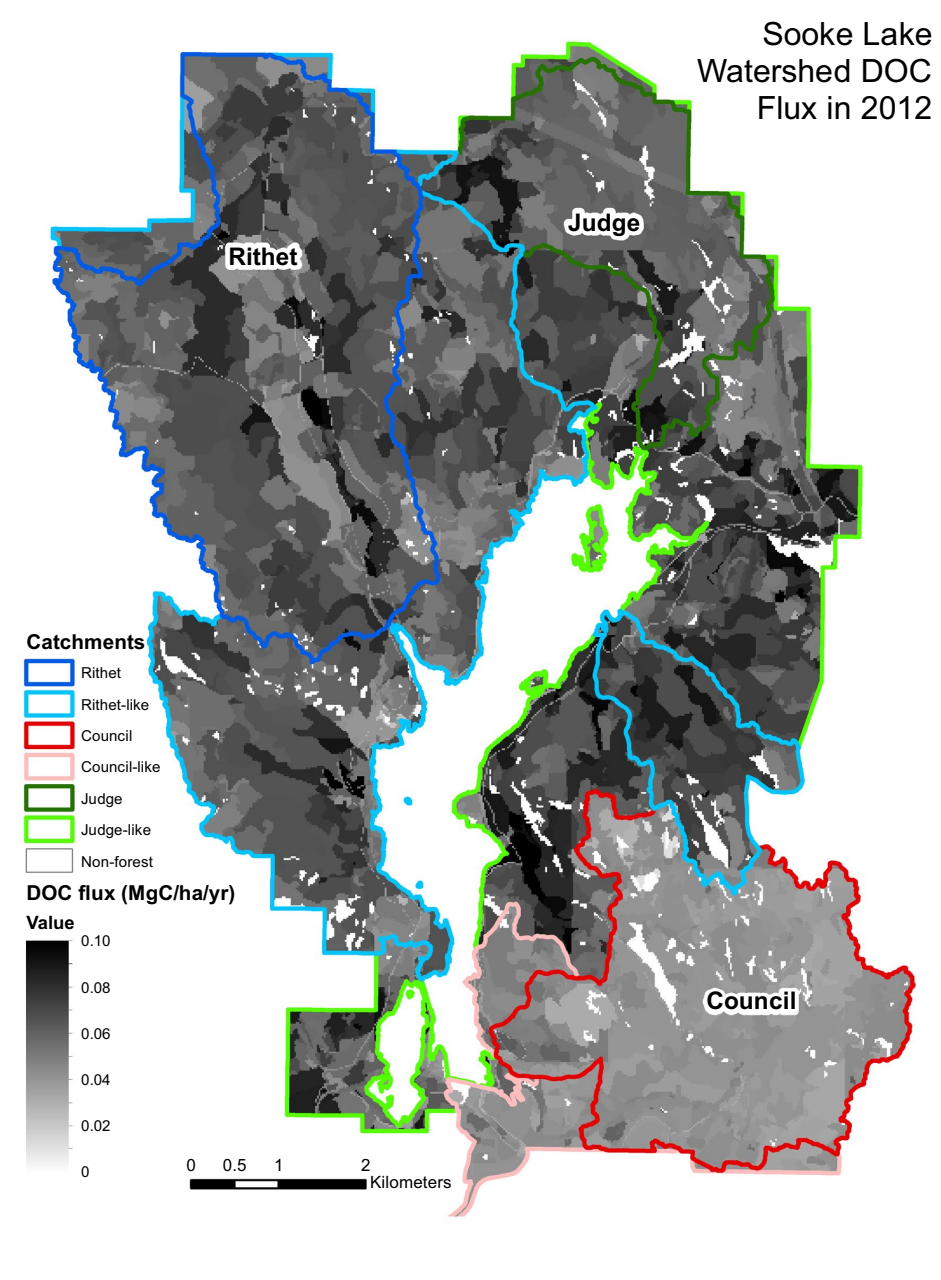
Sooke Lake Watershed DOC flux in Mg C ha^−1^ year^−1^ in 2012 and catchment delineation

The DOC fraction/fraction to atmosphere parameter values for the gauged catchments were applied to the ungauged catchments based on physiographic and hydrologic similarities [29] and model runs conducted for the entire SLW. Combined Rithet and Rithet-like catchments made up the largest proportion of the modelled area (Table 1); while, the area of the combined judge and judge-like catchments was greater than the Council and council-like catchments. Stands with high DOC fluxes in 2012 (Fig. 4) had higher soil C stocks and tended to be older. Areas west and south of Sooke Lake typically had lower DOC fluxes compared to forests east and northeast of the lake. The non-gauged catchments have differing amounts of C in the slow above and belowground DOM pools compared to the gauged catchments. As the ungauged catchments were assigned DOC parameters based on their hydrologic and physiographic characteristics and not on similar DOM pool sizes, the ha^−1^ DOC flux values differed slightly from those of the gauged catchments (Table 5). Significantly higher DOC fluxes were observed from polygons that recently had forests greater than 300 years on highly productive sites.

**Table 5:** CBM-CFS3 dissolved organic carbon (DOC) flux from slow aboveground (AG) and belowground (BG) DOM pools from 1996 to 2012 for -gauged and ungauged catchments and Sooke Lake Watershed totals

Landscape unit	Value	Mean	Max	Min	Total
Rithet + Rithet-like	AG	123.2	124.6	122.0	2094.3
	BG	24.7	24.9	24.5	419.5
	Total	147.9	149.6	146.5	*2513.8*
	ha^−1^	0.0377	0.0381	0.0373	0.6402
Council + Council-like	AG	14.5	14.8	14.2	246.2
	BG	9.3	9.4	9.2	158.2
	Total	23.8	24.0	23.6	*404.4*
	ha^−1^	0.0168	0.0169	0.0166	0.2745
Judge + Judge-like	AG	89.1	90.6	88.0	1515.4
	BG	18.1	18.4	17.8	307.2
	Total	107.2	109.0	105.8	*1822.6*
	ha^−1^	0.0380	0.0386	0.0375	0.6457
Watershed total	AG	75.6	124.6	14.2	3855.8
	BG	17.4	24.9	9.2	884.9
	Total	93.0	149.6	23.6	*4740.7*
	ha^−1^	0.0308	0.0386	0.0166	0.5766

All totals in Mg C or Mg C ha^−1^

For the calibration period, the average DOC flux from the terrestrial area of the SLW was 93.0 Mg C year^−1^ (0.0308 Mg C ha^−1^ year^−1^) with 81% of that coming from the slow aboveground DOM pool. Total DOC export for this period was 4740 Mg C. Over the 100-years historic period, 30,657 Mg C was exported from the terrestrial system via DOC, representing the upper bounds for what could be sequestered in lake sediment in this watershed.

### Land management scenarios carbon budgets

**Fig. 5:**
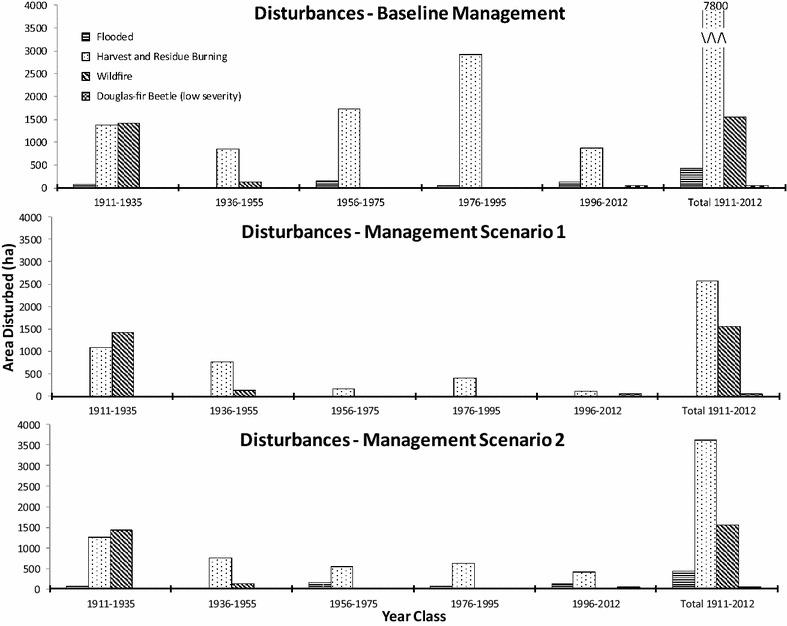
Disturbances by period for baseline, scenario 1 and scenario 2 management regimes

**Fig. 6:**
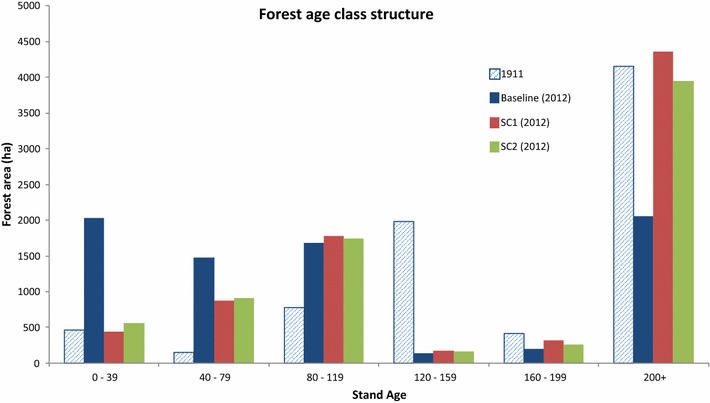
Forest age class structures in 1911 and 2012 for baseline, scenario 1 and scenario 2 management regimes

Three distinct periods of management are apparent in the baseline scenario. Until the mid-1950s, only a few large disturbances occurred. As these disturbances were mostly outside of the original CRD ownership the forest disturbances for this period are mirrored in the other scenarios, with the exception of the absent flooding event in SC1 (Fig. 5). The period of sustained yield forestry began after 1955 when clearcut, and residue burning and thinning events start to vary among the three scenarios and influenced forest age class structure in 2012. Both SC1 (4360 ha) and SC2 (3947 ha) had considerably more forest greater than 200 years than the Baseline scenario (2057 ha) (Fig. 6). In 2012, over 3500 ha were less than 80 years in the Baseline vs only 1306 ha and 1472 ha for SC1 and SC2, respectively. The third management period is denoted by the cessation of logging activity in the Baseline scenario in the mid-1990s and the resulting recovering in C stocks.

**Fig. 7:**
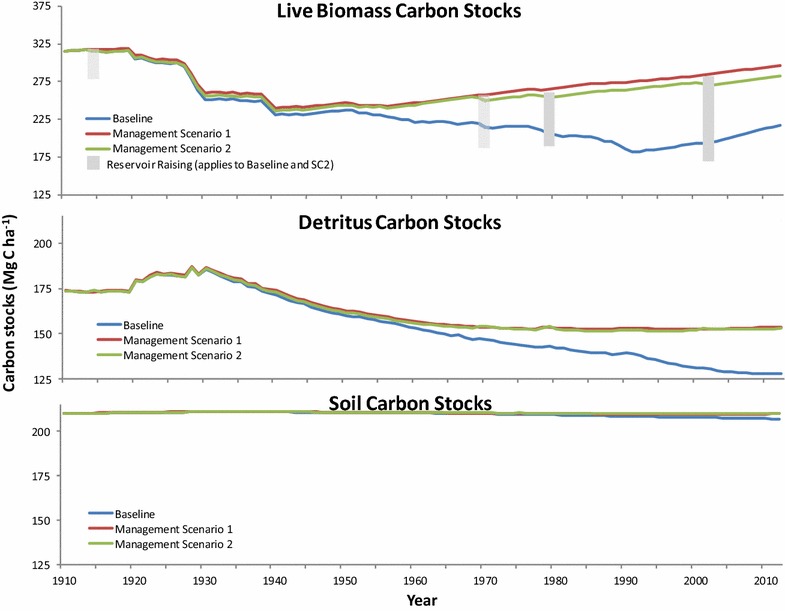
Baseline and alternative management scenario live biomass, detritus and soil C stocks 1911–2012

**Fig. 8:**
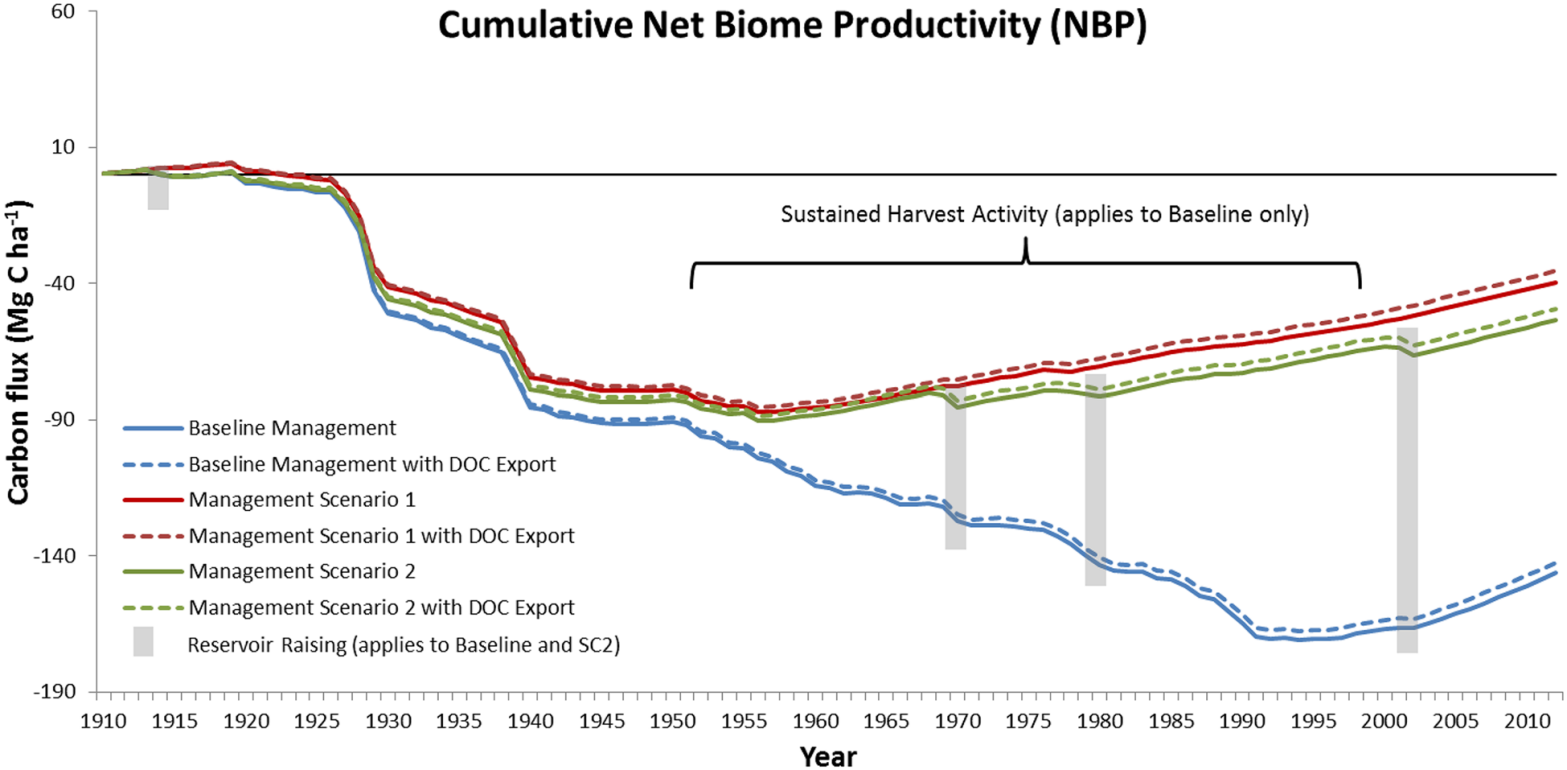
Cumulative net biome productivity with and without DOC as a carbon export mechanism

Comparisons among scenarios in live biomass C (above- and below-ground), detritus (litter and deadwood) and soil C stocks over the historical period are shown in Fig. 7. Because of the inherent stability of the soil C pools, differences due to management scenario were minimal over the study period), ranging between 2.8 and 3.1 Mg C ha^−1^ by 2012 (Table 6). Detritus stocks exhibited more differences, with SC2 and SC1 25.1 and 26.0 Mg C ha^−1^ greater than the Baseline scenario by 2012 (Table 6). Post-1960, detritus C stocks stabilize in SC1 and SC2 while in the Baseline they continue to decline until the end of the study period (Fig. 7). Live biomass stocks in all three scenarios began to recover after 1940 from a low between 231.0 Mg C ha^−1^ (baseline) and 240.0 Mg C ha^−1^ (SC1) (Fig. 7). However, by the mid-1950s, the recovery in stocks began to diverge, with SC1 and SC2 continuing to accumulate biomass whereas the Baseline scenario declined until the early-1990s. In 1991, the differences in biomass C for Baseline vs SC1 and SC2 scenarios reached a high of 93.5 and 83.5 Mg C ha^−1^, respectively, and then narrowed by 2012 (Fig. 7; Table 6). NBP describes the overall ecosystem C exchange of a landscape over multi-decadal time spans [41], and includes the removal of C due to disturbances [20]. Figure 8 shows the cumulative NBP (ƩNBP) for the three management regimes and the influence that DOC export has on the C budget. In the first 15 years ƩNBP remained approximately C neutral for all three scenarios. In areas outside CRD tenure, the large removals of live biomass C through HWP export and release to the atmosphere from slash burning resulted in a watershed-wide decline to −98.7 (baseline), −83.2 (SC1), and −85.8 (SC2) Mg C ha^−1^ in 1955 when including DOC export (ƩNBP^DOC^). All scenarios were approximately 2.0 Mg C ha^−1^ lower without DOC export. ƩNBP^DOC^ of the SC1 and SC2 scenarios began to recover after 1955, whereas the Baseline scenario continued to decrease. ƩNBP^DOC^ of SC1 and SC2 remained within ~10.0 Mg C ha^−1^ of one another until the mid-1960s when deforestation for reservoir expansion in 1970, 1980 and 2002 in SC2 increased HWP exports, and no biomass regrew on deforested lands. ƩNBP^DOC^ for the Baseline scenario began to recover in 1994 from a low of −167.4 Mg C ha^−1^ (−170.7 Mg C ha^−1^ ƩNBP) to its current (2012) level of −142.4 Mg C ha^−1^ (−146.2 Mg C ha^−1^ ƩNBP). In contrast, SC1 did not decline below −85.0 Mg C ha^−1^ (1956) and recovered to −35.4 Mg C ha^−1^ by 2012. The ƩNBP^DOC^ for SC2 was also at its lowest point in 1956 (−88.5 Mg C ha^−1^). While deforestation events in SC2 did dampen the ability to recuperate C losses from earlier in the century, ƩNBP^DOC^ had recovered to −49.4 Mg C ha^−1^ by 2012. Not unexpectedly, total HWP export for SC2 and SC1 were 46 and 60% lower, respectively, as compared to the Baseline of 882,746.2 Mg C (Table 6). More HWP was exported from SC2 than SC1 due to activities related to reservoir expansion and road access.

**Table 6:** Baseline, scenario 1 and scenario 2 carbon stocks and fluxes as of 2012

Flux/pool	Management scenarios		
	Baseline	Scenario 1	Scenario 2
Cumulative NBP (Mg C ha^−1^)			
No DOC export	−146.2	−39.4	−53.4
DOC export	−142.4	−35.2	−49.4
Carbon stocks (Mg C ha^−1^)			
Live biomass	217.4	296.1	282.0
Detritus	127.8	153.9	152.9
Soil C	207.1	209.9	210.2
Cumulative DOC export (Mg C ha^−1^)			
Aboveground slow	3.2	3.4	3.3
Belowground slow	0.7	0.7	0.7
Total	3.9	4.1	4.0
Total DOC export (Mg C) (1911–2012)	30,657.2	32,819.5	32,047.2
Total round wood export (Mg C) (1911–2012)	882,746.2	354,247.0	475,183.9

Cumulative DOC (ƩDOC) export was greatest in SC1 (4.1 Mg C ha^−1^) (Table 6) as the DOM stocks which feed DOC export in the model increased with the higher proportion of mature forest. As well, the lack of deforestation meant DOM stocks that were removed from the land base in the Baseline and SC2 management regimes were maintained in SC1 and continued to decay and release DOC. The SC1 and SC2 management scenarios could have potentially sequestered slightly higher amounts (2162 and 1390 Mg C, respectively). The impacts of forestry (Baseline vs SC2) and deforestation of reservoir expansion (SC1 vs SC2) on the land base (excluding Lot 87 and the Council Creek catchment) are further illustrated by the differences among scenarios in the spatial distribution of forest ecosystem C stocks in 2012 values (see Additional file 2: Figure S2 for total forest ecosystem C stocks in 2012 for Baseline, SC1 and SC2 across the SLW).

## Discussion

### Watershed scale DOC fluxes

Rithet summer stream flow is thought to be sourced from a small bedrock aquifer [29, 41], since there are no snowpacks, glaciers or significant lakes to contribute to summer discharge. Groundwater can also be a source of high DOC [42]. While Kenny [43] investigated aquifer extent across the CRD, little is known about the geological formations and their porosity and permeability within the SLW. Therefore, groundwater DOC input into the reservoir was not considered in the watershed DOC fluxes.

Modelling the C exported from the terrestrial to the inland aquatic system on a watershed scale suggests allochthonous C storage in lake sediment may be a significant C sink. Physiographic differences, specifically percent area of wetlands and lakes, forest cover age structure (Table 2), and size of slow above and belowground DOM pools were the primary terrestrial forces driving long term DOC export to fluvial systems. The inundation of littoral wetlands areas due to reservoir raising events can also have a significant impact on the nutrient loading within a lake, generally [44, 45] and in Sooke reservoir in particular [46]. However, the impact of reservoir expansion on terrestrial-to-aquatic DOC transfers was not included in this study, and therefore reported DOC flux values to the reservoir may be an underestimation in this respect. The slow DOM pools and selected DOC fraction parameters capture well the trend and magnitude of long term DOC loads observed in the gauged catchments. Long term trends in DOC load increases have been observed in areas of western and northern Europe, most likely due to acid deposition histories resulting from industrial development [47, 48]. The current configuration of the CBM-CFS3 does not include a mechanism to model the short term (1–5 years) event-driven spikes in DOC load due to effects of disturbance on stream DOC concentrations. On some forested landscapes hydrologic events (i.e., storms and snowmelt) can be the source of approximately 86% of terrestrially-derived DOC to the aquatic environment [10]. If more mobile sources of DOM (i.e., litter) are available due to disturbances such as forest harvesting or wildfire then this terrestrially-sourced DOC will be magnified initially and then be depleted. The introduction of a DOC fraction parameter to another, more mobile C pool (i.e., the aboveground very fast DOM) or a transfer function built into the disturbance matrices might improve the ability of the model to simulate the short term DOC export that would occur after disturbance.

DOC fraction parameters must be calibrated based on the physiographic and hydrological characteristics of the study area in question. Differences in DOC transfer rates are highly variable spatially and sensitive to temperature and resulting decomposition rates. Study area-specific mean annual temperature could increase the accuracy of the soil decomposition rates compared to the ecozone normals used in this study. The impact of precipitation on DOC fluxes is considerable as well. In similar sized ocean-draining watersheds on the central coast of British Columbia, where annual rainfall is double and forest soils are thicker organic layers and have higher soil C contents than that observed in the SLW [49], DOC fluxes (0.377 Mg C ha^−1^ year^−1^) are almost 10 times those estimated in this study [50]. The annualized DOC flux parameters selected for the three catchment types only represent a small fraction of the slow above and belowground DOM pools; however, accumulation over many years could impact the C sequestration expectations, and therefore the watershed-scale C budget [10].

While the question of DOC fate was beyond the scope of this study, the final destination of terrestrially-sourced C is an important component of coupled terrestrial–inland aquatic modelling efforts. Dean and Gorham [51] estimated that average long-term C burial rates of lakes of 14 g C m^−2^ year^−1^, with reservoirs sequestering on average a much higher amount (400 g C m^−2^ year^−1^). The upper bounds of annual carbon burial for the SLW may be up to 37 g C m^−2^ year^−1^; integrating CO_2_ respired from the reservoir will adjust this figure downward. Average DOC concentrations from the Sooke reservoir spillway were lower than those recorded for streams draining the three gauged catchments (Sooke Reservoir: 2.43 mg C/l; Judge: 5.67 mg C/l; Rithet: 3.47 mg C/l; Council: 3.43 mg C/l). While within-lake C fixation through aquatic gross primary production is considered to be a net source of C, the addition of terrestrially-sourced C into the system, which can be equal to or greater than autochthonous C [52], can potentially accumulate and result in long term C storage in lake sediments [53]. Thus, the increased reservoir area and sediment deposition resulting from reservoir creation could, over time, potentially offset the sudden release of C that occurs during deforestation from reservoir expansion.

Potential increases in the frequency and magnitude of rainfall events with a changing climate may result in increased DOC export to the Sooke reservoir and this reinforces the need for more consistent DOC monitoring in order to inform adaptation strategies. Dore et al. [54] reported that precipitation patterns have changed since monitoring began in the SLW in 1914. The IPCC predicts that in the Pacific Northwest and Western Canada, the variance in seasonal precipitation will increase and temperatures will rise steadily over the next century [55]. Drier summer soils, changes in decomposition rates and more rapid, intense flushes of DOC through higher intensity rainfall events could have water quality implications.

### Possible reservoir expansion effects on methane fluxes

An important consideration in both terrestrial and aquatic C cycling is the significance of methane (CH_4_) because of its role as a potent GHG which can affect the intensity of global climate change. As a GHG, CH_4_ is 28 times more potent than CO_2_ [56]; this fact coupled with the speed at which it is accumulating in the atmosphere relative to CO_2_, averaging 1% per year over the last few decades [57], makes it an important component to study in terrestrial-inland aquatic ecosystems. The major natural source of CH_4_ stems from methanogenesis which mainly occurs in wetlands and wet lowland areas where C is released from wetland and lakebed sediments [57]. In upland regions, a small amount of CH_4_ is absorbed into the soil by methanotrophic bacteria, although this is only a fraction of what is released from lowland areas [57]. CH_4_ cycling in forest ecosystems can also be impacted by many forestry practices such as land clearing (for quarries, roads, etc.) and nitrogen fertilization which have been found to produce nitrite that persistently inhibit methanotrophic bacteria [57].

### Relative impacts of deforestation and forest management

**Fig. 9:**
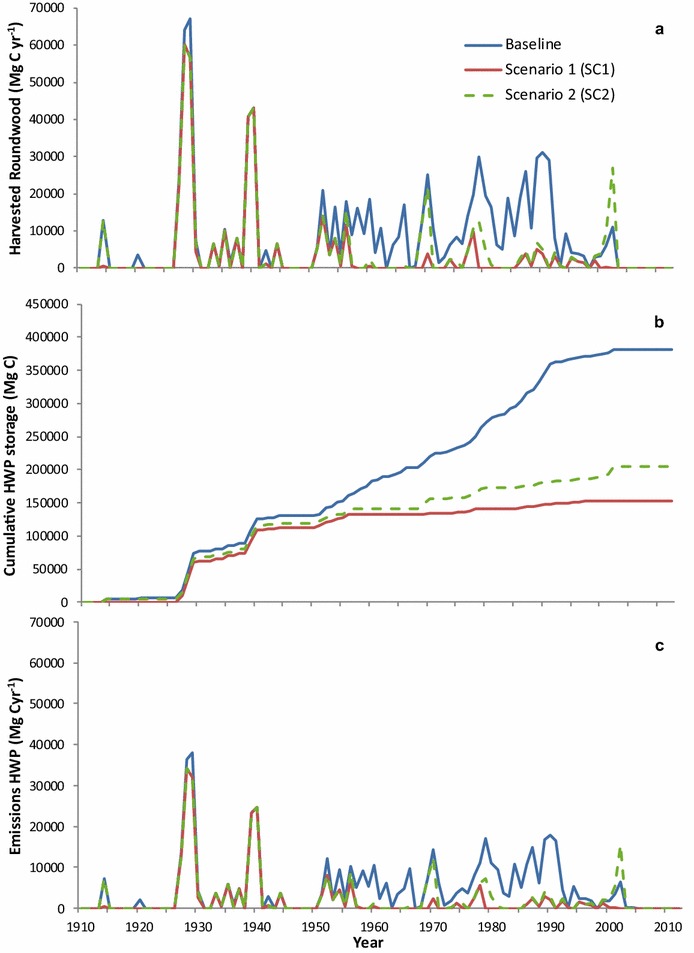
Baseline, scenario 1 and scenario 2 harvested round wood (**a**), cumulative storage in harvested wood products (**b**) and emissions from processing (**c**) from 1911–2012

The multiple reservoir raisings had a stepped effect on ƩNBP over the study period (Fig. 8). At the watershed scale, the impact of deforestation (SC1 vs SC2) resulted in a cumulative decrease of approximately 14.0 Mg C ha^−1^ by 2012 equivalent to 110,991 Mg C less being sequestered. In contrast, sustained yield forestry activity within the CRD’s tenure (Baseline vs SC2) accounts for a 93.0 Mg C ha^−1^ difference in ƩNBP by 2012, equivalent to 738,809 Mg C less being sequestered. This shows that while deforestation due to reservoir creation removes biomass stocks and ends forest C sequestration on those lands, over 100 years, the recurring removal of C in the form of harvested round wood (Fig. 9a) had a substantially greater impact on the landscape C budget than did reservoir creation. That said, the removal of C from the SLW during forestry operations is partially offset by renewed sequestration after stands establish and tree growth resumes.

For different ecosystems, and different scales of analysis a mix of forest management techniques is more likely to optimize forest C sequestration [3]. Man et al. [58] explored two general forest management methods for increasing C sequestration and found that strategies that reduced harvest levels had greater C sequestration benefits than strategies that increased growth. In the SLW, the harvest reduction strategy exhibited in SC2 whereby the CRD-owned land becomes a reserve shows a stark increase in C stored in biomass pools in comparison with the Baseline.

### Accounting for exported round wood in harvested wood products

The preceding analysis assumes all C in exported round wood (IPCC rules until 2012) is emitted to the atmosphere and forgoes accounting for C stored in HWP, the cumulative difference of which is 176,222 Mg C (Fig. 9b). Emissions associated with HWP (Fig. 9c) are 2.5 and 1.8 times greater in the Baseline scenario than in SC1 and SC2, respectively, but accounting for the fate of HWP enables a commensurate fraction of C to be stored in products. Considering current harvest rotation ages of less than 50 years in some managed forests [59], the residency time of C in manufactured products could in fact be longer than that sequestered in managed forests.

Managing forests for conservation purposes often increases the C stocks on the land base; however, the risk of natural disturbance (e.g., wildfire, drought, insects or disease) means the ecosystem C storage can be at risk [4]. In their case study Man et al. [58] found that greater than 25% stand mortality can nullify the C storage gains from the reserved forest in the short-term, while 50% stand mortality has a permanent negative effect. Using a forest reserve strategy whereby an area is removed from the harvesting land base might have detrimental impacts on ecosystem C storage due to unforeseen natural disturbances or climate change impacts on decay rates. Projected future changes of natural disturbance patterns call into question the effectiveness of existing forest management mechanisms to achieve C sequestration objectives [20].

Different forest management regimes can have a considerable impact on forest C biomass and DOM stocks, especially when these management decisions are compared over decadal and longer time scales. In BC, current C credit legislation dictates that C credits may not be granted unless the atmospheric effect of the C removals endures for a minimum of 100 years [60]. This requires that the effects of management decision must be considered, at minimum, on a multi-decadal scale. The comparison of SC1 and SC2 with the 100-year Baseline C budget of the SLW enables the C budget effect of the specific management decisions that led to deforestation for reservoir creation as well as sustained forest harvest to be quantified. Also, the Baseline C budget allows for future extrapolation of C stocks and C fluxes. While CBM-CFS3 implicitly includes environmental differences through temperature input, and growth curve manipulation, it does not explicitly integrate the potential effects of climate change into growth, decay or decomposition rates. Work is progressing to investigate environmental change effects on forest ecosystem carbon stocks [61, 62]. Changing growth and decomposition dynamics observed in the Pacific Northwest over the twentieth century [63] need to be integrated to examine CBM-CFS3′s ability to model the effects of climate change on future forest ecosystem C budgets.

## Conclusions

DOC flux from temperate forest ecosystems is spatially complex and a small but persistent C flux which may have long term implications for C storage in inland aquatic systems. CBM-CFS3 parameterization of DOC flux from the SLW forest ecosystem used [DOC] and stream flow measurements (1996–2012) from three catchments. Model calibration yielded three distinct DOC transfer fractions from the aboveground slow pool resulting in DOC fluxes between 0.0154 and 0.0381 Mg C ha^−1^ year^−1^.

When applied to the entirety of the SLW, the modelled accumulation of DOC from uplands sources totalled 30,657 Mg C for the 100 year period. While we do not assert all fluvial transported C remains within the inland aquatic system in the long term, our estimate represents an upper bound for what could be sequestered through burial in reservoir/lake sediment for this watershed. This is a first step to integrating fluvial transport of C into a forest carbon model by parameterizing DOC flux from the detrital and soil C pools.

Employing alternative management scenarios is an effective means of understanding how past management decisions influence current and future C stocks and fluxes. By 2012, deforestation due to reservoir creation and expansion resulted in the watershed sequestering 14 Mg C ha^−1^ less than it otherwise would have with no deforestation. Sustained harvest activity had a substantially greater impact with sequestration reduced by an additional 93 Mg C ha^−1^. However as approximately half of the round wood C removed during logging ends up in wood products, over 176,000 Mg C could have remained in storage and out of the atmosphere reducing the cumulative impact of forestry activity from 93 to 71 Mg C ha^−1^.

While successive deforestation related to reservoir expansion does influence watershed-scale C budgets, over multi-decadal time periods, sustained harvest activity was more impactful in the SLW. Understanding the role forest ecosystems play in the global C cycle and, more specifically, integrating the aquatic components of those landscapes into modelling efforts will enable a more accurate determination of anthropogenic impacts on the C cycle.

## Additional files

**Additional file 1: Figure S1.** Daily stream flow and dissolved organic carbon (DOC) concentration, measured and simulated, for Rithet, Judge and Council catchments 1996–2012.

**Additional file 2: Figure S2.** Total forest ecosystem C stocks in 2012 for Baseline Scenario 1 and Scenario 2 across the Sooke Lake watershed.

## Abbreviations

C: carbon
GHG: greenhouse gas
BC: British Columbia
CBM-CFS3: Carbon Budget Model of the Canadian Forest Service
CO2: carbon dioxide
CH4: methane
IPCC: Intergovernmental Panel on Climate Change
SLW: Sooke Lake Watershed
DOC: dissolved organic carbon
ha: hectares
yr: years
CRD: Capital Regional District
AMLE: study adjusted maximum likelihood estimation
AIC: Akaike information criterion
OLS: ordinary least squares
DOM: dead organic matter
GPG: good practice guidance
HWP: harvested wood products
NBP: net biome production
SC1: alternative management scenario #1
SC2: alternative management scenario #2
