# Supplementary figures and images for: Historical effects of dissolved organic carbon export and land management decisions on the watershed-scale forest carbon budget of a coastal British Columbia Douglas-fir-dominated landscape
Source: Carbon Balance Manag. 2017 Jul 14;12:15. doi: 10.1186/s13021-017-0083-z (PMC5509570; doi:10.1186/s13021-017-0083-z)

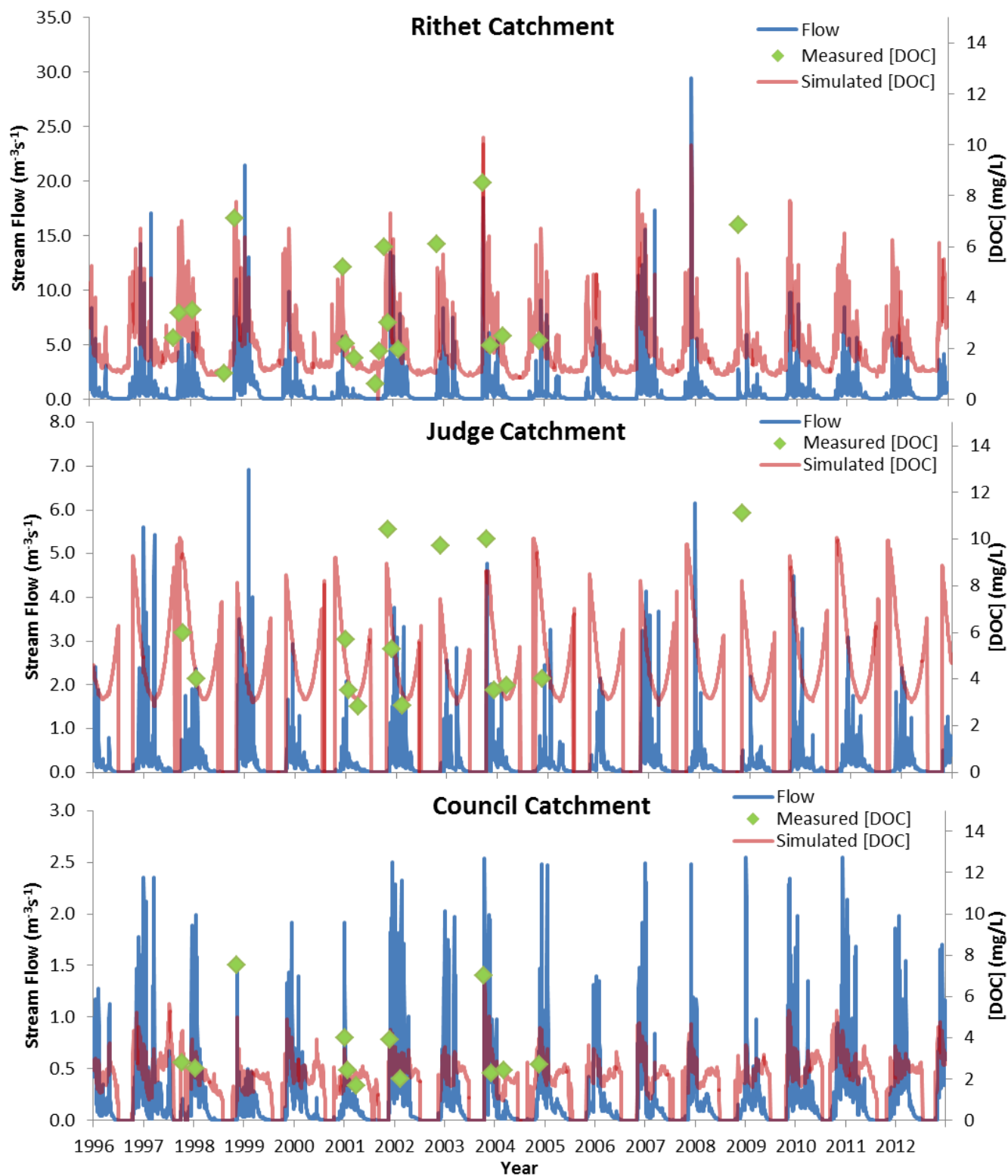

Supplement: Supplementary file 1 — Additional file 1: Figure S1. Daily stream flow and dissolved organic carbon (DOC) concentration, measured and simulated, for Rithet, Judge and Council catchments 1996–2012. [file 13021_2017_83_MOESM1_ESM.pdf]

Baseline

SC1

SC2

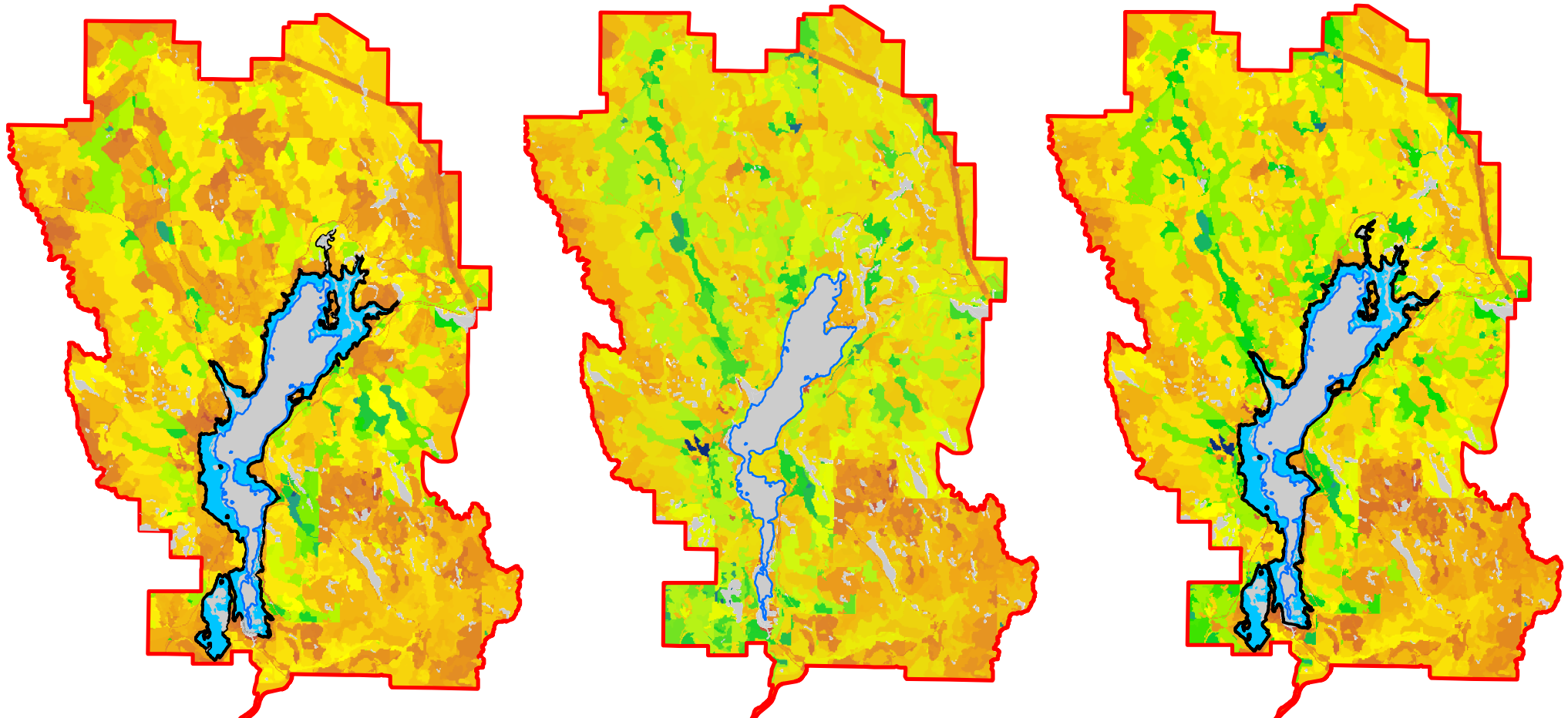

**Forest Ecosystem C stocks** **Boundaries**

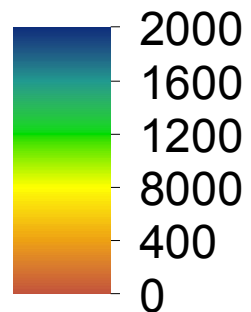

**Mg C/ha**

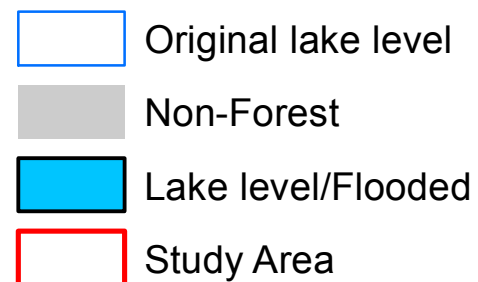

Supplement: Supplementary file 2 — Additional file 2: Figure S2. Total forest ecosystem C stocks in 2012 for Baseline Scenario 1 and Scenario 2 across the Sooke Lake watershed. [file 13021_2017_83_MOESM2_ESM.pdf]
